# Simulations of γ-Valerolactone Solvents and Electrolytes for Lithium Batteries Using Polarizable Molecular Dynamics

**DOI:** 10.3390/molecules30020230

**Published:** 2025-01-08

**Authors:** Adriano Pierini, Valentina Migliorati, Juan Luis Gómez-Urbano, Andrea Balducci, Sergio Brutti, Enrico Bodo

**Affiliations:** 1 Department of Chemistry, Sapienza University of Rome, P. le Aldo Moro 5, 00185 Rome, Italy; valentina.migliorati@uniroma1.it (V.M.); sergio.brutti@uniroma1.it (S.B.); 2 Institute for Technical Chemistry and Environmental Chemistry, Friedrich-Schiller University, Philosophenweg 7a, 07743 Jena, Germany; juanlu.gomez.urbano@uni-jena.de (J.L.G.-U.); andrea.balducci@uni-jena.de (A.B.); 3 Center for Energy and Environmental Chemistry (CEEC), Friedrich-Schiller University, Philosophenweg 7a, 07743 Jena, Germany; 4 CNR-ISC—Consiglio Nazionale Delle Ricerche, Istituto dei Sistemi Complessi, 00185 Rome, Italy; 5 GISEL—Centro di Riferimento Nazionale per i Sistemi di Accumulo Elettrochimico di Energia, 50121 Florence, Italy

**Keywords:** γ-valerolactone, electrolytes, lithium batteries, molecular dynamics, polarizable forcefields

## Abstract

In this paper, we present a molecular dynamics study of the structural and dynamical properties of γ-valerolactone (GVL) both as a standalone solvent and in electrolyte formulations for electrochemistry applications. This study involves developing a new parameterization of a polarizable forcefield and applying it to simulate pure GVL and selected salt solutions. The forcefield was validated with experimental bulk data and quantum mechanical calculations, with excellent agreement obtained in both cases. Specifically, two 1M electrolyte solutions of lithium bis(fluorosulfonyl)imide and lithium bis(oxalate)borate in GVL were simulated, focusing on their ionic transport and highlighting ion solvation structure. Ion pairing in the electrolytes was also investigated through enhanced sampling molecular dynamics, obtaining a detailed picture of the ion dynamics in the GVL solution.

## 1. Introduction

Since the introduction of commercial lithium-ion batteries (LIBs) in the 1990s, extensive research has been conducted to develop improved electrolytes, with a focus not only on enhancing performance but also on advancing safety, sustainability and cost-effectiveness [1,2,3]. It has become mandatory, in fact, to rethink materials for the development of energy storage devices with attention to their sustainability, environmental impact, renewability and, possibly, integration within a circular economy framework.

**Figure 1:**
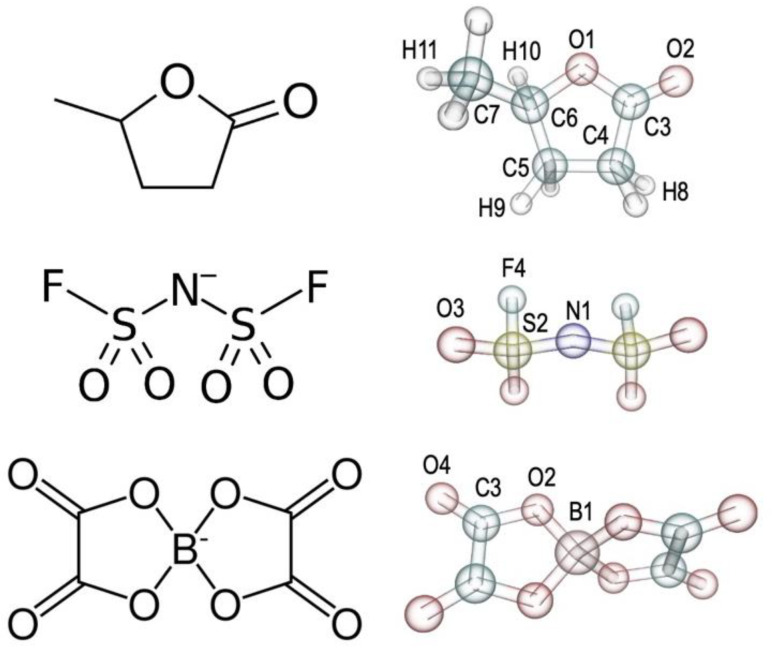
Molecular structures and forcefield indexing of γ-valerolactone (**top**), bis(fluorosulfonyl)imide anion (**center**) and bis[oxalato]borate anion (**bottom**).

γ-Valerolactone (GVL, dihydro-5-methyl-2(3*H*)-furanone) (see Figure 1) is a promising platform molecule with the potential to satisfy the above requirements [4]. GVL is a biodegradable, naturally occurring, non-toxic chemical that can be used as a solvent for biomass processing, and as a food and fuel additive [5,6]. In fact, it can be directly synthesized from biomass sources, specifically materials such as cellulose or hemicellulose [7,8]. This allows for low environmental impact and, potentially, unexpensive large-scale production from abundant and renewable feedstock [9]. Moreover, its biodegradability [10] and low toxicity [7] support the fundamental quest for more environmentally compatible materials (see Refs. [5,11] for a discussion of the eco-compatibility and sustainability of GVL synthesis and use in energy-based applications).

The physicochemical properties of GVL also make it an interesting alternative solvent for liquid organic electrolytes. Table 1 compares the properties of GVL and propylene carbonate (PC), one of the most used organic carbonates in electrochemistry, which shares a very similar chemical structure. A wide liquid range and high relative permittivity are important requirements for electrochemistry and battery applications. Thanks to its electric dipole moment (4.71 D [12]), GVL should provide a sufficient solubility of the conductive salt at the typical 1 mol·L^−1^ concentration [13]. Also, a wide electrochemical window has been reported for GVL [14], with a higher anodic limit than PC, which could prove essential in affording stability in high-voltage devices. GVL has been investigated and employed as a green solvent for many organic reactions [15]. Another promising potential application of GVL is its use as a solvent for the manufacture of electrodes, as a bio-based replacement for conventional NMP-based processes [16,17]. However, the first applications of GVL as a nonaqueous medium for electrochemical energy storage were only devised very recently. GVL-based electrolytes for electric double-layer capacitors have shown good thermal and electrochemical stability, as well as transport properties [18]. Similarly, a 1M solution of lithium bis(oxalate)borate (LiBOB) in GVL, with high stability and good conductivity, has demonstrated excellent performance in lithium-ion capacitors. The electrochemical stability of GVL-based electrolytes can differ significantly depending on the conductive salt employed. However, in most cases, an electrochemical stability window larger than 5.0 V vs. Li^+^/Li was measured (5.1 and 5.5 V vs. Li^+^/Li for LiTFSI and LiBOB salts, respectively) [19,20]. This extended voltage window (comparable to that of reference electrolyte systems) allows the utilization of GVL-based electrolytes in combination with a wide range of cathode materials, such as LFP or NMC. Furthermore, the suitable recycling of GVL through sustainable water-based methods was demonstrated, which further enhance the sustainability aspect of this bio-based solvent [20].

Among the conductive salts employed to formulate GVL-based electrolytes, LiBOB and LiTFSI present appealing properties for their application in energy storage devices. Unlike standard salts, such as LiPF6, the strong resistance toward hydrolysis of LiTFSI not only prevents the formation and release of toxic subproducts (e.g., HF and LiF) [21], but also enables its recovery through simple and non-toxic water-based approaches [22]. On the other hand, the fluorine-free nature of LiBOB reduces the burden associated with the mining of raw materials needed to produce conventional salts, and it stands out due to its ability to simultaneously protect the aluminum current collector of the positive electrode from anodic dissolution and create a robust solid electrolyte interphase on the surface of negative electrodes [23,24]. These enhanced safety and sustainability features of LiBOB and LiTFSI make them promising candidates for next-generation energy storage devices, especially when combined with the bio-derived GVL solvent.

In this work, we present a theoretical study of the GVL liquid and its electrolyte solutions for lithium battery applications. For this purpose, we developed a polarizable forcefield for performing molecular dynamics (MD) simulations, following the established AMOEBA model. The inclusion of electrostatic polarization in classical forcefields is highly recommended for systems with a large degree of ionicity such as electrolytes [25,26,27,28,29,30,31]. It generally provides an improved description of structure and diffusivities when compared to fixed-charge models, avoiding the tedious and repetitive empirical tuning of forcefield parameters to match specific target properties. The validation of the forcefield was carried out by a direct comparison of the simulated bulk properties of liquid GVL with available experimental data and by a vis-à-vis match of the forcefield intermolecular energy profile with gas-phase ab initio calculations. We report results based on the following: (i) a structural analysis of short-range molecular ordering in pure GVL; (ii) the structural and transport properties of two electrolyte formulations in GVL, one with lithium bis(fluorosulfonyl)imide (LiFSI) and the other with LiBOB; and (iii) a thermodynamic study of the salt dissociation in GVL using metadynamics (umbrella sampling) in a simplified diluted system.

**Table 1:** Selected physicochemical properties of γ-valerolactone and propylene carbonate. ε is relative permittivity (at 293 K); ρ is liquid density (at 298 K); η is shear viscosity (at 298 K).

Property	GVL	PC
T_melt_/°C	−31 ^a^	−50 ^b^
T_boil_/°C	206 ^a^	242 ^b^
T_flash_/°C	96 ^c^	116 ^c^
ε	36.9 ^d^	66.1 ^e^
ρ/g·cm^−3^	1.05 ^f^	1.20 ^g^
η/cP	1.86 ^h^	2.51 ^g^

^a^ From Ref. [11]; ^b^ from Ref. [32]; ^c^ from Ref. [33]; ^d^ from Ref. [34]; ^e^ from Ref. [35]; ^f^ from Ref. [36]; ^g^ from Ref. [37]; ^h^ from Ref. [38].

## 2. Results and Discussion

### 2.1. Forcefield Validation

The forcefield parametrization procedure involved three molecules/ions: GVL and the FSI^−^ and BOB^−^ anions. The parameters of the Li^+^ cation were already available in the standard AMOEBA forcefield, and we used them without modifications. The new forcefield parameters, together with those already present in the AMOEBA forcefield, are reported in the Supplementary Materials in Tinker format.

**Figure 2:**
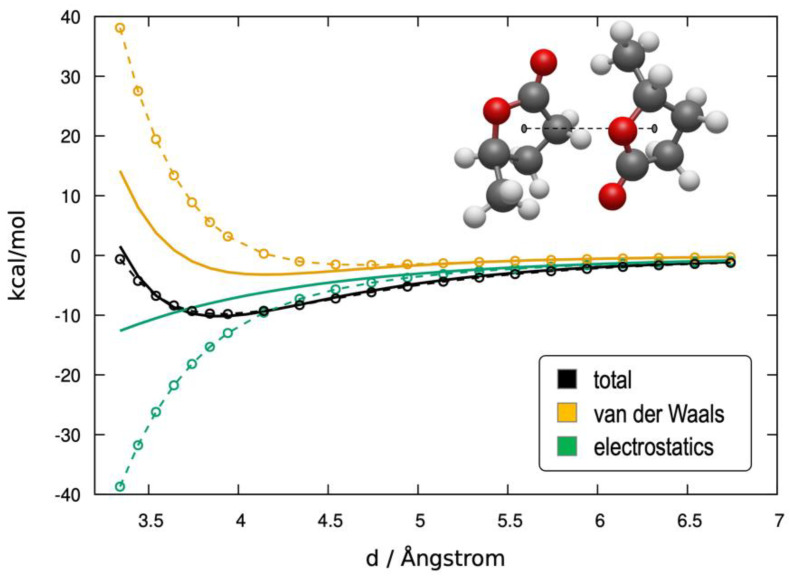
Potential energy scan of an interacting GVL-GVL dimer. The x coordinate represents the distance between the two centers of mass (the equilibrium dimer geometry is 3.74 Å). Full lines are forcefield energies; dashed lines are reference ab initio energies calculated with SAPT2.

The first validation of the forcefield consisted of testing the reliability of the parametrization in reproducing intermolecular potentials. As an example, Figure 2 reports a comparison of the forcefield interaction energy (solid black line) with that obtained from the ab initio potential energy (dashed black line) of two interacting GVL molecules. The ab initio total interaction energies are perfectly reproduced along the whole range of coordinates, maintaining excellent accuracy also in short separation. The energy decomposition analysis provides details on the electrostatic and van der Waals components of this energy. It shows that the forcefield, at short range, underestimates both the repulsive van der Waals component (yellow) and the attractive electrostatic one (green) as obtained by SAPT2. This is not entirely unexpected, because when the two centers of mass are at short range, a subset of the interatomic distances become small, and both the forcefield model and the SAPT2 partitioned energies are less accurate [39]. A similarly good agreement in interaction energy is also found for the anion–solvent pairs, whose potential energy scans are reported in Figure S2.

The accuracy of the intermolecular potential calculated by us can also be appreciated through the comparison of predicted bulk GVL properties with experimental data. The computed and measured evaporation enthalpy, density and self-diffusion coefficient are reported in Table 2. The bulk liquid cohesion energy is correctly predicted by our forcefield, which reproduces the enthalpy of evaporation within 3%. The molecular packing is also correctly predicted, with an error for the estimated density below 1%. Moreover, the self-diffusion coefficients calculated with MD are in reasonable agreement with the ones measured by PGF-NMR, showing that the forcefield electrostatics can properly describe dynamical properties as well as structural ones.

**Table 2:** Properties of liquid γ-valerolactone at 293 K from MD simulation and from experimental data. ρ is the density; ∆H_vap_ is the enthalpy of vaporization; D_self_ is the self-diffusion coefficient.

Property (at 293 K)	MD	Experiment
ρ/g·cm^−3^	1.06	1.05 ^a^
∆H_vap_/kcal·mol^−1^	12.5	12.9 ^b^
D_self_/10^−10^ m^2·^s^−1^	9.8	8.1 ^c^

^a^ From Ref. [36]; ^b^ from Ref. [40]; ^c^ from Ref. [41] (measured at 303 K).

### 2.2. Pure Liquid Structure

**Figure 3:**
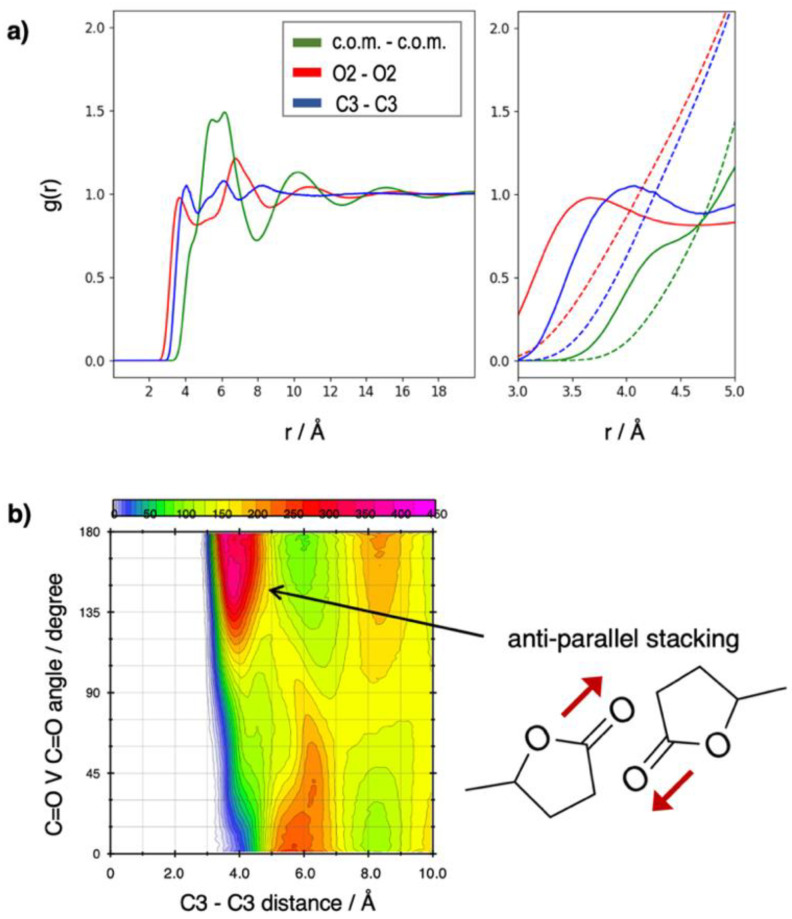
Structural analysis of the pure GVL liquid simulation: (**a**) intermolecular RDFs of the centers of mass (green), carbonyl oxygens (red) and carbonyl carbons (blue); the panel on the right is an enlargement where we represent as the running integration numbers of the RDFs as dashed lines; (**b**) combined distribution function of the intermolecular C=O/C=O vector angles and the intermolecular carbonyl carbon distances.

Figure 3a reports the intermolecular radial distribution function (RDF) in pure GVL. The position of the first peak in the carbonyl O–O and C–C RDFs (red and blue solid lines), along with the corresponding running integration numbers (red and blue dashed lines), indicates the formation of dimers led by carbonyl–carbonyl interactions. This interaction driven by dipole–dipole forces is the source of the structuring of the fluid that one can see in the RDF between the GVL centers of mass (green solid line).

The dashed lines in the right panel of Figure 3a show that at 4.5 Å, the running integral of the carbonyl O–O and C–C RDFs is already larger than 1, thereby indicating that the dipole–dipole interaction is responsible for the clustering of several GVL molecules.

The nature of the short-range order in GVL is highlighted by the data in Figure 3b, which reports the combined distribution function obtained from the reciprocal orientation of the C=O dipoles (as the angular distribution function of two vectors lying along the two C=O bonds) and the distance between carbonyl groups (C–C RDF). The intense red region, centered around 4 Å and between 140° and 180°, shows that the first-neighbor interaction is a GVL dimer where the two carbonyl groups are in anti-parallel alignment (thus maximizing the dipolar electrostatic interaction). Inversely, the region with the second-highest density, corresponding to the second peak in the C–C RDF around 6 Å, indicates a parallel arrangement of the carbonyl groups. The next maximum at 8 Å is again associated with anti-parallel orientation. Such findings indicate that the short-range structure consists of shells induced by alternating patterns of orientations of the carbonyl dipoles. This result is supported by previous spectroscopic reports [42] and is in substantial agreement with similar findings of preferential anti-parallel dimer formation in liquid ethylene [43], propylene [44] and butylene carbonates [45].

### 2.3. Solvation and Conductivity in Electrolytes

To characterize the properties of electrolytes based on concentrated salts in GVL, 1M solutions of LiFSI and LiBOB were simulated at a temperature of 293 K. The calculated properties are reported in Table 3. To check for the goodness of the electrolyte simulations, the density and conductivity calculated for LiBOB/GVL can be compared with the values of 1.14 g·cm^−3^ and 4.9 mS·cm^−1^, respectively, measured by Teoh at al [19]. Moreover, the lower conductivity values calculated for LiBOB vs. LiFSI (3.1 and 8.5 mS·cm^−1^, respectively) align well with those reported for carbonate-based electrolytes [46,47].

Given the range of σ values, GVL is classified as a good solvent for high-conductivity liquid electrolytes. The lithium transport number *t*_+_, which is also reported in Table 3, expresses the fraction of current that is carried by lithium ions and is given as

(1) $$t_{+}=\frac{\sigma_{+}}{\sigma_{+}+\sigma_{-}}=\frac{D_{+}}{D_{+}+D_{-}}$$

where we assume the limit of high dilution.

The calculated values of Table 3 are in the typical range of common organic liquid electrolytes [48] and are in agreement with the experimental data for conductivities and densities specifically measured for these formulations. The difference between the computed densities and the measured ones is only 1.7%, while that for conductivities is larger. This is not unexpected since the computational conductivities are computed here using the ionic diffusions and assuming an ideal diluted solution (Equation (1)), while the measured ones come from solution impedance (which includes non-ideal effects). As shown previously [49,50], the non-ideality of a solution can impact the calculated values of bulk conductivities even if the ionic diffusion coefficients are reproduced correctly. In general, computing conductivities is a difficult task for simulations, although the use of polarization in the present instance has certainly contributed to the clear prediction of both the correct order of magnitude and the trend across the two electrolytes (this is often not the case for non-polarizable forcefields, e.g., see Ref. [25]).

**Figure 4:**
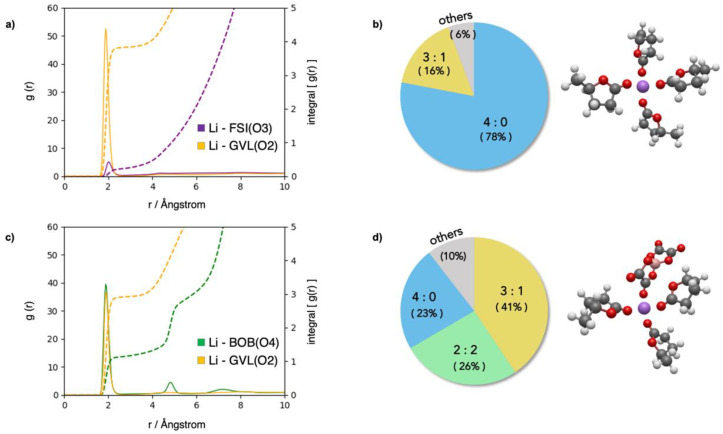
Analysis of lithium coordination in the 1M LiFSI (**a**,**b**) and 1M LiBOB (**c**,**d**) electrolytes. (**a**–**c**): RDFs of the cation–solvent and cation–anion interactions (solid lines) and their running integration numbers (dashed lines). (**b**–**d**): statistical probabilities of different compositions of the coordination shell around Li^+^, showing also the most probable molecular structure on the right side; the labels on the pie chart indicate the composition of the coordination shells as n_solvent_:n_anion_ ratios.

We find in our case that the trends of σ and *t*_+_ are in reverse order for the two electrolytes, with 1M LiFSI being the most conductive but displaying a lower transport number: 0.37 vs. 0.46 for 1M LiBOB. This difference stems from the mobility of the anions and may be partly related to their different solvation environments in the two electrolytes. Figure 4 shows the Li^+^-GVL and Li^+^–anion RDFs for the 1M LiFSI (Figure 4a) and the 1M LiBOB (Figure 4c) solutions. The values of the running integration numbers of the RDFs (dashed lines) up to the first minimum tell us the coordination numbers of the two ligands in the first solvation shell around the Li^+^ cations. For the 1M LiBOB in GVL solution, the two plateaus after 2 Å in Figure 4c (green and yellow dashed lines) show a 3:1 ratio between coordinating GVL molecules and BOB anions. On the other hand, for the 1M LiBOB in LiFSI formulation (Figure 4a), a dominant contribution is observed from GVL molecules only. Hence, the much larger extent of ion pairing for the LiBOB salt can explain both its lower conductivity (with respect to LiFSI) and the predicted difference in Li^+^ transport numbers. In fact, as the motion of cations and anions in LiBOB is strongly correlated through the formation of stable ion pairs, a large fraction of the ions would contribute to the ratio in Equation (1) with similar D_+/−_ values, with *t*_+_ therefore approaching a value of one half.

The pie charts in Figure 4b,d show how the different molecular environments around Li^+^ cations contribute to the average coordination numbers; here, the most statistically abundant configurations of the first solvation shell are expressed as solvent-to-anion ratios (the most probable one is depicted on the right of the respective chart). The analysis shows that for LiBOB, the 3:1 solvent–anion ratio arising from the RDF is the most likely configuration of the Li^+^ solvation shell, although there are significant contributions from the 4:0 and 2:2 types of configurations. This implies that the isolated Li^+^ is not negligible and, at the same time, that multi-anion coordination is also present and may impact ionic aggregation.

The LiFSI electrolyte shows a very different conformation. Most (~80%) of the Li^+^ is dissociated from the anionic counterpart, and only a minor fraction of ionic couples survives in a 3:1 configuration.

### 2.4. Study of Ion Pairing

**Figure 5:**
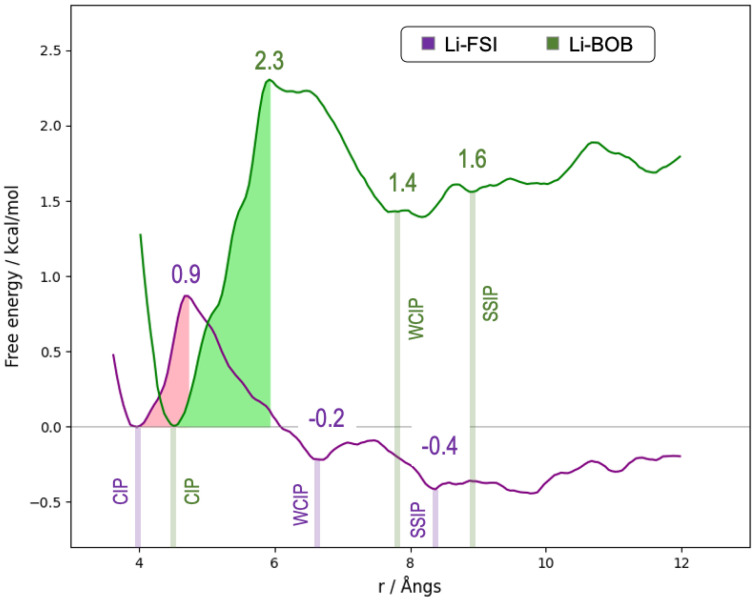
Helmholtz free energy of dissociation of LiFSI (purple line) and LiBOB (green line) ion pairs in GVL, calculated by umbrella sampling. Filled areas indicate the energy barriers which must be overcome to dissociate the contact ion pair. Vertical lines indicate the sampling windows where CIP, WCIP and SSIP (see text) are identified.

In order to understand the dissociation dynamics of LiFSI/LiBOB salts in GVL solutions, we evaluated the free energy involved in the dissociation of ionic couples. To this aim, we used an ideal configuration where the salt was infinitely diluted that allowed us to disentangle the thermodynamics of salt dissociation from the complex network of ion–ion interactions that characterize concentrated electrolyte solutions. Figure 5 shows the Helmholtz free energy landscapes sampled in the canonical NVT ensemble.

**Figure 6:**
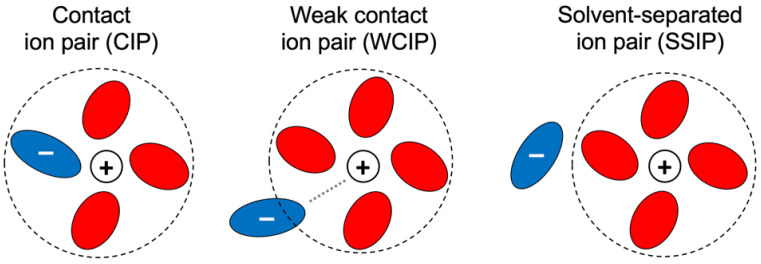
Schematic representations of the contact, weak contact and solvent-separated ion pairs. Cations and anions are indicated in white and blue; solvent molecules are red.

The reaction coordinate is calculated as the distance between lithium and the center of mass of the anion. For both salts, the zero in energy is set to the first minimum of the curve, which corresponds to the free energy of the contact ion pair (CIP). In this configuration, the four-fold coordination of Li^+^ is saturated by three solvent molecules and one anion, all presenting mono-coordination toward the cation (see Figure 6).

Moving away from the CIP configuration, a free energy barrier appears, which is the energy cost necessary to separate the ionic partners. At room temperature, the barriers are rather low (0.9 kcal/mol for FSI and ~2.3 kcal/mol for BOB), thus indicating that ion pairing/unpairing dynamics in GVL are rather fast.

At a distance of around 9 Å, the ion pairs with both anions appear to be essentially dissociated in what are usually referred to as solvent-separated ion pairs (SSIPs; see Figure 6), where the electrostatic cation–anion interaction is screened by the layer of solvent. At a distance farther than 10 Å, a plateau is reached where the free energy fluctuates only slightly, as the anion moves further away into the secondary solvation shell of lithium. The free energy difference from the CIP to the SSIP is slightly negative for LiFSI but is positive for BOB, where the SSIP lies 1.6 kcal/mol above the CIP. Given that the kinetic barriers are low enough to allow fast equilibration, this thermodynamic difference is the reason behind the large gap in the ionicity of the two 1M solutions.

In both free energy profiles, an intermediate energetic minimum can be spotted right after the main energy barrier and before the SSIP plateau. At these geometries, the cation–anion separation is somewhat midway between that of the CIP and that of the SSIP, and the ionic interaction is still strong enough to compensate for the distortion of the ideal four-molecule solvation around the fully dissociated cation, causing a solvation geometry which we refer to as a weak contact ion pair (WCIP; see Figure 6) whose energy lies quite close to the energy of the SSIP.

The specific nature of these WCIP configurations is reported in Figure S3. At a distance corresponding to the WCIP (6.4 Å for LiFSI and 7.8 Å for LiBOB), the anion is not entirely outside the first solvation shell, and the oxygen–lithium RDF still presents a defined interaction peak that, although less intense than in the CIP, is very different from the structureless shape in the SSIP. This feature is equally identifiable in both systems, despite the largely different shapes of the free energy profiles. The ability to form such weak contact ion pairs of lithium salts could therefore be expected to be common to many GVL solvent–salt systems.

Owing to the data in Figure 5, focusing on the values of the energetic barriers that the ionic couple must overcome to separate, one can easily see how the separation of the anion and the cation is a facile pathway for LiFSI (0.9 kcal/mol), even at modest temperatures. On the other hand, separating Li^+^ from BOB^−^ requires 2.3 kcal/mol of free energy that, albeit still not prohibitive, indicates an enhanced likelihood for the ionic couple of remaining bound at low temperatures. It is worth noting (see Figure 5) that the dissociation of LiFSI is a weakly spontaneous process due to a small but negative free energy balance (ΔA < 0), while this is not the case for LiBOB. It is straightforward to trace the difference in the persistence of ionic couples back to the bulk result of conductivity in the two electrolytes, where effective charge transport is enhanced by the presence of solvated, charged ions and hampered by the persistence of ionic couples.

## 3. Methods

The AMOEBA forcefield [51] parameters were obtained from isolated molecule calculations following the procedure in the reference paper of the code Poltype2 [52], which we already applied successfully in previous works [49,53,54]. Bonded interactions (except for torsion angles, which were fitted in the present work), atomic polarizabilities and Van der Waals parameters were taken from an already existing AMOEBA forcefield. The electrostatic multipoles were instead obtained by fitting them to newly computed MP2/aug-cc-pVTZ electron densities evaluated at the respective optimized geometries (see Refs. [55,56,57] for a discussion of the method’s accuracy in evaluating geometries and electrostatic properties). The parametrized intermolecular potentials were tested by comparison with ab initio calculations and, specifically, energy perturbation analysis of selected interacting molecular pairs. These were obtained with SAPT2 [58] calculations that were run on the open-source Psi4 code [59] using an aug-cc-pVDZ basis set.

The molecular dynamics simulations were run with the program Tinker-HP [60]. Details of the cell boxes built for the pure solvent and the binary electrolyte simulations are reported in Table S1. Our simulation protocol consisted of energy minimization, followed by a 2 ns NPT run with a fixed temperature (293 K) and pressure (1 bar) using the Bussi thermostat and the Berendsen barostat, respectively. An equilibration was then run for 5 ns in NVT conditions, and the trajectory data were finally collected for 6 additional ns, storing the atomic coordinates every 50 ps.

The MD trajectories were used to calculate thermodynamic and dynamical properties. The enthalpy of evaporation was obtained using the standard formula [61]:

(2) $$∆H_{\mathrm{vap}}=E_{\mathrm{gas}}+E_{\mathrm{liq}}+RT$$

where *E*_gas_ only includes the intramolecular energy contributions to energy, and *E*_liq_ is the potential energy of the simulated cell. The Nernst–Einstein equation [62] was used to evaluate the ionic conductivity:

(3) $$\sigma =\frac{e^{2}\;}{VK_{b}T}\sum_{j}N_{j}z_{j}^{2}D_{j}$$

where *N_j_*, *z_j_* and *D_j_* are, respectively, the number, charge and diffusional coefficients of anions and cations. *D_j_* is calculated as the time-averaged mean-square displacement of the molecular or atomic centers of mass:

(4) $$D_{j}=\underset{t\to \infty }{\lim}\frac{1}{6Nt}\sum_{i}\langle {\left[r_{i}(t)-r_{i}(0)\right]}^{2}\rangle$$

To explore the free energy profile for salt dissociation, umbrella sampling along the Li+–anion center of mass distance was performed by subdividing the dissociation coordinate into windows of 0.2 Å, while imposing a harmonic bias potential of 40 kcal·mol^−1^, restraining the distance. This value is relatively high, but nevertheless, it allowed the proper sampling of the reaction coordinate, as can be seen in the distributions reported in Figure S1. The umbrella sampling calculations along the anion–cation distance were performed on two simulation cells of reduced size, containing only one ionic pair and 133 solvent molecules. In each window, an NVT equilibration was run at 293 K for 500 ps, and production data were accumulated for an additional 300 ps. The free energy profile was then reconstructed using the weighted histogram analysis method [63] (WHAM) implemented in the code by A. Grossfield [64].

### Physicochemical Measurements

The solvent GVL (Sigma Aldrich, Taufkirchen, Germany, ≥99%) was dried prior to utilization over molecular sieves (3 Å), and a water content <20 ppm was confirmed by Karl Fischer titration (C20 Coulometric KF Titrator, METTLER TOLEDO, Zurich, Switzerland). LiBOB (eNovation, Green Brook, NJ, USA, 98%) and LiFSI (Solvionic, Toulouse, France, 99.9%) salts were employed to prepare the electrolytes. The ionic conductivity of the electrolytes was measured with a Modulab XM ECS potentiostat (AMETEK Scientific Instruments, Oak Ridge, TN, USA). Conductance was determined using the alternating current resistance of the electrolyte placed between parallel platinum electrodes with a known cell constant.

## 4. Conclusions

We have presented a new set of results stemming from molecular dynamics simulations aimed at understanding the dynamics and structure of liquid electrolytes based on γ-valerolactone, a green solvent which holds promise in radically improving the environmental sustainability of organic electrolytes for battery manufacturing. We have used a new polarizable forcefield that we parameterized for the neutral GVL molecule and the BOB anion.

Through comparison with high-level ab initio calculations and bulk liquid experimental data, we have verified that the polarizable electrostatic model employed by us can reliably reproduce intermolecular interactions for both solvent–solvent and solvent–anion pairs, suggesting that the present forcefield could be easily transferable to other compositions. The simulations of the pure GVL solvent, whose calculated bulk properties are in excellent agreement with their experimental counterparts, highlighted the peculiar features in the local structure of the liquid, which revealed a clear short-range order dominated by strong dipolar interactions. Binary electrolytes with 1M LiFSI or LiBOB salts were also simulated, showing a strong dependence between the ionicity of the solution and its molar conductivity. In particular, the LiFSI/GVL electrolyte, which presents a large extent of salt dissociation, was predicted to have a remarkably high ionic conductivity of about 8–9 mS·cm^−1^ at 20 °C. The different speciation of the Li^+^ solvates in the two electrolytes was further investigated by studying the thermodynamics of ion pairing in solution. Umbrella sampling simulations for both salts showed that the pairing dynamics are essentially dominated by their free energies of dissociation, owing to low kinetic barriers toward separation. Moreover, partially separated cation–anion pairs were found to have energy close to that of solvent-separated ion pairs along the dissociation coordinate, which can significantly impact the solvation/desolvation processes occurring during Li^+^ transport across the electrolyte and to the electrode surface.

These results, derived from theoretical calculations, enhance the understanding of the fundamental physicochemical behavior of GVL in solutions, possibly helping in the rational design of GVL-based electrolytes and compatible battery materials.

## Figures and Tables

**Table 3:** Properties of 1M electrolytes at 293 K from MD simulations. ρ is the density; *D*_+_, *D*_–_ and D_s_ are the self-diffusion coefficients of the cation, the anion and the solvent, respectively; σ is the ionic conductivity; *t*_+_ is the transport number of Li^+^ ions.

Property (at 293 K)	GVL + LiBOB		GVL + LiFSI	
	MD	Expt	MD	Expt
ρ/g·cm^−3^	1.16	1.14	1.17	1.15
*D*_+_/10^−10^ m^2·^s^−1^	0.37		0.52	
*D*_–_/10^−10^ m^2·^s^−1^	0.44		0.90	
D_s_/10^−10^ m^2·^s^−1^	0.95		1.32	
σ/mS·cm^−1^	3.1	4.90	8.5	6.86
*t*_+_	0.46		0.37	

## Data Availability

The raw data supporting the conclusions of this article will be made available by the authors upon reasonable request.

## Supplementary Materials

The following supporting information can be downloaded at https://www.mdpi.com/article/10.3390/molecules30020230/s1. Table S1: Detailed composition of the simulation cells; Figure S1: RDFs of Li-anion distance in each window of the umbrella sampling; Figure S2: Potential energy scans of the anion-solvent pairs; Figure S3: Illustration of CIP (top panels), WCIP (middle panels) and SSIP (bottom panels) from umbrella sampling; Forcefield parameters in Tinker format.
