# Supplementary material for: Simulations of γ-Valerolactone Solvents and Electrolytes for Lithium Batteries Using Polarizable Molecular Dynamics
Source: Molecules. 2025 Jan 8;30(2):230. doi: 10.3390/molecules30020230 (PMC11768070; doi:10.3390/molecules30020230)
Supplement: Supplementary file 1 [file molecules-30-00230-s001.zip › molecules-3403423-supplementary.pdf]

Simulations of γ-Valerolactone Solvents and Electrolytes for Lithium Batteries Using Polarizable Molecular Dynamics

Adriano Pierini[1]*, Valentina Migliorati[1], Juan Luis Gómez-Urbano[2,3], Andrea Balducci[2,3], Sergio Brutti[1,4,5], Enrico Bodo[1]*

1 Department of Chemistry, Sapienza University of Rome, P. le Aldo Moro 5, 00185 Rome, Italy
2 Institute for Technical Chemistry and Environmental Chemistry, Friedrich-Schiller University, Philosophenweg 7a, 07743 Jena, Germany
3 Center for Energy and Environmental Chemistry (CEEC), Friedrich-Schiller University, Philosophenweg 7a, 07743 Jena, Germany
4 CNR-ISC—Consiglio Nazionale Delle Ricerche, Istituto dei Sistemi Complessi, 00185 Rome, Italy
5 GISEL—Centro di Riferimento Nazionale per i Sistemi di Accumulo Elettrochimico di Energia, 50121 Florence, Italy
* Correspondence: adriano.pierini@uniroma1.it (A.P.); enrico.bodo@uniroma1.it (E.B.)

Supplementary Material

1. Methods details: simulation compositions, umbrella sampling

Table S1. Detailed composition of the simulation cells: number of total atoms, number of molecules, salt concentration (mol·L^{-1}) and molar fraction.

System	N. atoms	N. molecules (solvent)	N. molecules (salt)	[Li^+]	$\chi(Li^+)$
**GVL**	15990	1066	0	-	-
**GVL + 1M LiFSI**	16000	1000	100	0.98	0.091
**GVL + 1M LiBOB**	15997	975	98	0.97	0.091

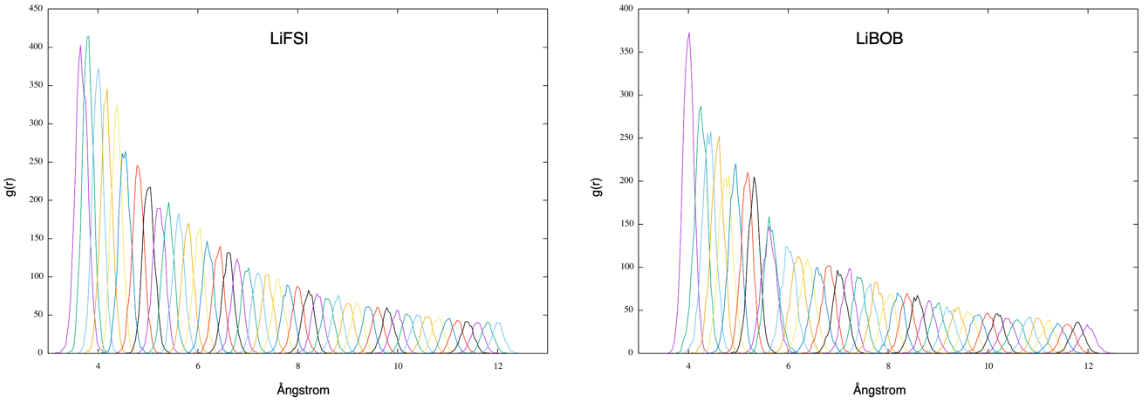

Figure S1. RDFs of Li-anion distance in each window of the umbrella sampling. A regular distribution of evenly separated peaks with overlapping tails typically indicates that the reaction coordinate has been properly sampled.

2. Validation of the force field using SAPT method for FSI-GVL and BOB-GVL

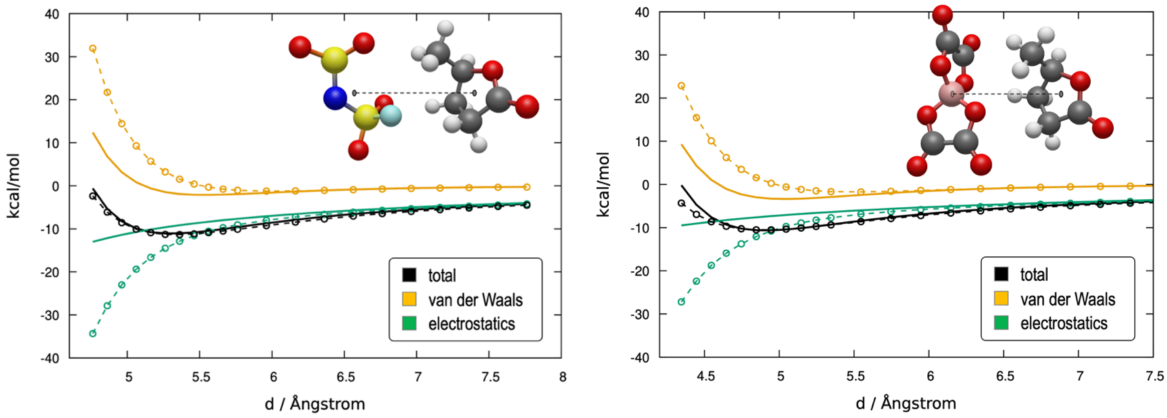

Figure S2. Potential energy scans of the anion-solvent pairs: FSI-GVL (top) and BOB-GVL (bottom). The x axis represents the distance between the two centers of mass. Full lines are forcefield energies, dashed lines are reference ab-initio energies calculated with SAPT.

3. More details on the free energy calculations. An illustration of the ionic couple states along the dissociation process.

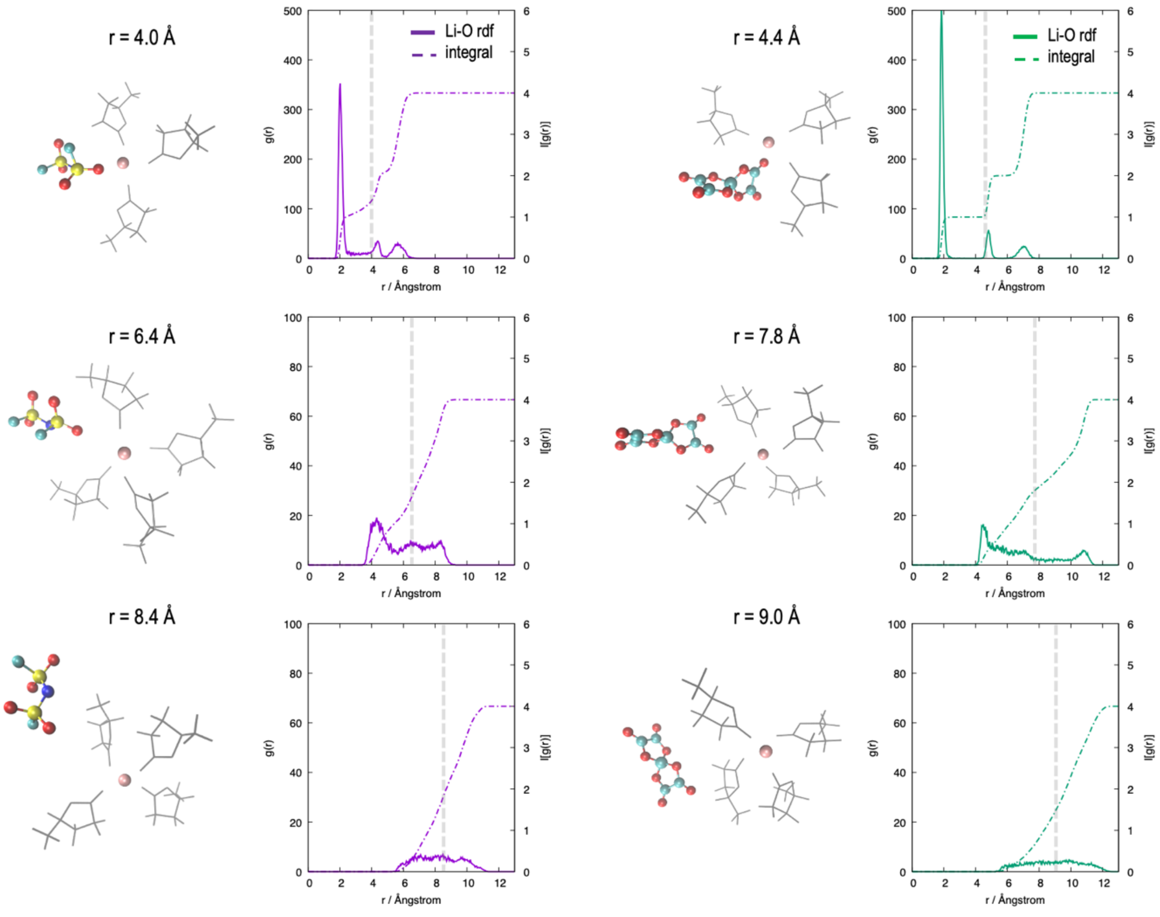

Figure S3. Illustration of CIP (top panels), WCIP (middle panels) and SSIP (bottom panels) from umbrella sampling. The geometrical structures are extracted from the trajectory frames of the corresponding sampling window. Solvent molecules are sketched as grey sticks, ions as sticks-and-balls models. The corresponding RDFs of the lithium-oxygen distance and its integral are also reported.

Forcefield parameters in Tinker format

```
# gamma-valerolactone (GVL)

atom        1    1   O     "gvl O1"                    8    15.999    2
atom        3    3   C     "gvl C3"                    6    12.011    3
atom        2    2   O     "gvl O2"                    8    15.999    1
atom        4    4   C     "gvl C4"                    6    12.011    4
atom        5    5   C     "gvl C5"                    6    12.011    4
atom        6    6   C     "gvl C6"                    6    12.011    4
atom        7    7   C     "gvl C7"                    6    12.011    4
atom        8    8   H     "gvl H8"                    1     1.008    1
atom        9    9   H     "gvl H9"                    1     1.008    1
atom       10   10   H     "gvl H10"                   1     1.008    1
atom       11   11   H     "gvl H11"                   1     1.008    1

vdw        10       2.870    0.024    0.910
vdw         7       3.820    0.101    0.000
vdw         5       3.820    0.101    0.000
vdw         4       3.820    0.101    0.000
vdw        11       2.900    0.022    0.900
vdw         9       2.960    0.024    0.920
vdw         8       2.980    0.024    0.940
vdw         6       3.650    0.101    0.000
vdw         2       3.300    0.112    0.000
vdw         1       3.405    0.110    0.000
vdw         3       3.780    0.106    0.000

bond        5    6     237.000    1.530
bond        4    5     205.640    1.520
bond        9    5     346.460    1.090
bond        7    6     244.210    1.510
bond        1    6     212.000    1.450
bond       10    6     352.510    1.100
bond       11    7     345.980    1.090
bond        1    3     309.380    1.370
bond        2    3     778.350    1.210
bond        4    3     213.750    1.520
bond        8    4     346.460    1.090

angle       5    6    1      93.340  105.510
angle       5    6    7      97.840  114.050
angle       9    5    6      48.500  109.380
angle       5    4    8      49.200  113.770
angle       4    5    6      79.410  103.310
angle       9    5    4      49.200  112.410
angle      10    6    5      52.790  110.110
angle       3    4    5     100.960  104.080
angle       9    5    9      29.000  108.330
anglep      1    3    2      61.740  122.480
angle      11    7    6      72.290  110.180
angle       3    1    6      94.740  110.240
angle       1    6    7      69.120  108.400
angle      10    6    7      31.070  110.520
angle      11    7   11      36.400  108.750
angle       8    4    3      73.190  109.130
angle      10    6    1      56.730  107.180
anglep      4    3    1      59.630  108.970
anglep      4    3    2      53.030  128.550
angle       8    4    8      29.000  107.860

strbnd      5    6    1      27.930   27.930
strbnd      5    6    7       5.710    5.710
strbnd      9    5    6      27.930   27.930
strbnd      5    4    8      27.930   27.930
strbnd      4    5    6      27.930   27.930
strbnd      9    5    4      27.930   27.930
strbnd     10    6    5      27.930   27.930
strbnd      3    4    5      27.930   27.930
strbnd      1    3    2      16.920   16.920
strbnd     11    7    6      20.450   20.450
strbnd      3    1    6     -21.330  -21.330
strbnd      1    6    7      11.600   11.600
```

```
strbnd     10    6    7       5.710    5.710
strbnd     11    7   11       5.710    5.710
strbnd      8    4    3      27.930   27.930
strbnd     10    6    1      27.930   27.930
strbnd      4    3    1      16.920   16.920
strbnd      4    3    2      16.920   16.920
strbnd      8    4    8      27.930   27.930

opbend      1    3    0    0      0.000
opbend      2    3    0    0      0.000
opbend      4    3    0    0      0.000

torsion     2    3    1    6   -0.670 0.0 1  6.287 180.0 2  0.000 0.0 3
torsion     4    3    1    6   -0.670 0.0 1  6.287 180.0 2  0.000 0.0 3
torsion     3    1    6    7    0.854 0.0 1 -0.374 180.0 2  0.108 0.0 3
torsion     1    6    7   11    0.000 0.0 1  0.000 180.0 2  0.266 0.0 3
torsion     3    1    6    5    0.854 0.0 1 -0.374 180.0 2  0.108 0.0 3
torsion     1    3    4    5    0.854 0.0 1 -0.374 180.0 2  0.108 0.0 3
torsion     2    3    4    5    0.854 0.0 1 -0.374 180.0 2  0.108 0.0 3
torsion    10    6    1    3    0.000 0.0 1  0.000 180.0 2  0.108 0.0 3
torsion     4    5    6    1    0.854 0.0 1 -0.374 180.0 2  0.108 0.0 3
torsion     9    5    6    1    0.000 0.0 1  0.000 180.0 2  0.108 0.0 3
torsion     5    6    7   11    0.000 0.0 1  0.000 180.0 2  0.212 0.0 3
torsion     4    5    6    7    0.854 0.0 1 -0.374 180.0 2  0.108 0.0 3
torsion     9    5    6    7    0.000 0.0 1  0.000 180.0 2  0.108 0.0 3
torsion     9    5    6   10    0.000 0.0 1  0.000 180.0 2  0.299 0.0 3
torsion     3    4    5    6    0.854 0.0 1 -0.374 180.0 2  0.108 0.0 3
torsion     8    4    5    6    0.000 0.0 1  0.000 180.0 2  0.108 0.0 3
torsion    10    6    5    4    0.000 0.0 1  0.000 180.0 2  0.108 0.0 3
torsion     9    5    4    8    0.000 0.0 1  0.000 180.0 2  0.299 0.0 3
torsion     3    4    5    9    0.000 0.0 1  0.000 180.0 2  0.108 0.0 3
torsion    11    7    6   10    0.000 0.0 1  0.000 180.0 2  0.305 0.0 3
torsion     8    4    3    2    0.000 0.0 1  0.000 180.0 2  0.108 0.0 3
torsion     8    4    3    1    0.000 0.0 1  0.000 180.0 2  0.108 0.0 3

multipole    6 1 5       0.02549
                         0.16014  0.00000  0.18337
                        -0.12900
                         0.00000 -0.48867
                        -0.16164  0.00000  0.61767
multipole    7 6 1      -0.16666
                        -0.00275  0.00000  0.27390
                        -0.16693
                         0.00000 -0.23151
                         0.03261  0.00000  0.39844
multipole   11 7 6       0.07552
                        -0.00027  0.00000 -0.06660
                         0.00617
                         0.00000  0.00554
                        -0.00455  0.00000 -0.01171
multipole    5 6 4      -0.12873
                         0.32194  0.00000  0.27152
                         0.27923
                         0.00000 -0.45311
                        -0.12854  0.00000  0.17388
multipole    3 2 1       0.70464
                         0.26779  0.00000  0.23100
                         0.29052
                         0.00000 -0.16414
                        -0.13182  0.00000 -0.12638
multipole    1 3 6      -0.21127
                         0.51034  0.00000  0.20806
                         0.40393
                         0.00000 -0.68812
                         0.02907  0.00000  0.28419
multipole   10 6 1       0.07148
                        -0.01934  0.00000 -0.02000
                        -0.02625
                         0.00000  0.06996
                        -0.03426  0.00000 -0.04371
multipole    2 3 1      -0.64670
                         0.02270  0.00000 -0.11863
                        -0.36013
                         0.00000  0.16502
```

```
                         0.01992  0.00000  0.19511
multipole    4 3 5      -0.26673
                         0.33985  0.00000  0.21090
                         0.42250
                         0.00000 -0.33897
                        -0.14268  0.00000 -0.08353
multipole    9 5 6       0.08728
                        -0.00940  0.00000 -0.04369
                        -0.00434
                         0.00000  0.02166
                         0.00863  0.00000 -0.01732
multipole    8 4 3       0.10868
                        -0.01270  0.00000 -0.00790
                         0.00238
                         0.00000 -0.00593
                        -0.00454  0.00000  0.00355

polarize         6 1.6200 0.3900 10
polarize         7 1.4150 0.3900 11
polarize        11 0.4800 0.3900 7
polarize         5 1.4150 0.3900 9
polarize         3 1.6000 0.3900 1 2
polarize         1 0.8320 0.3900 3
polarize        10 0.4800 0.3900 6
polarize         2 0.9140 0.3900 3
polarize         4 1.4150 0.3900 8
polarize         9 0.4800 0.3900 5
polarize         8 0.4800 0.3900 4
```

```
# bis(fluorosulfonyl)imide (FSI)

atom        12    12   N     "fsi- N1"              7    14.007    2
atom        13    13   S     "fsi- S2"             16    32.066    4
atom        14    14   O     "fsi- O3"              8    15.999    1
atom        15    15   F     "fsi- F4"              9    18.998    1

vdw         13        3.910    0.385    0.000
vdw         12        3.710    0.110    0.000
vdw         15        3.220    0.061    0.000
vdw         14        3.400    0.100    0.000

bond        12   13     550.000    1.600
bond        13   15     250.000    1.630
bond        14   13     606.000    1.460

angle       12   13   14      75.000  113.590
angle       12   13   15      75.000   96.836
angle       13   12   13      65.000  115.680
angle       14   13   15      75.000  107.080
angle       14   13   14      80.000  118.310

strbnd      12   13   14       0.000    0.000
strbnd      12   13   15       0.000    0.000
strbnd      13   12   13       0.000    0.000
strbnd      14   13   15       0.000    0.000

torsion     14   13   12   13    5.589 0.0 1 -0.739 180.0 2 -2.732 0.0 3
torsion     15   13   12   13    0.698 0.0 1 -6.160 180.0 2  6.160 0.0 3

multipole    12 -13 -13    -0.68766
                            0.00000  0.00000  0.16212
                            0.44355
                            0.00000 -0.56592
                            0.00000  0.00000  0.12237
multipole    13 15 12       1.60194
                           -0.22035  0.00000  0.29756
                           -0.65446
                            0.00000  0.40432
                            0.10719  0.00000  0.25014
multipole    14 13 15      -0.67863
                           -0.00909  0.00000 -0.05194
                           -0.17801
                            0.00000 -0.26075
                            0.05189  0.00000  0.43876
multipole    15 13 12      -0.40085
                            0.07193  0.00000 -0.03719
                           -0.08417
                            0.00000 -0.21560
                            0.09578  0.00000  0.29977

polarize       12  1.4437 0.3900 13
polarize       13  2.9941 0.3900 12 14 15
polarize       14  0.8588 0.3900 13
polarize       15  0.3481 0.3900 13
```

```
# bis(oxalato)borate (BOB)

atom          16    16   B     "bob- B1"            5    10.811    4
atom          17    17   O     "bob- O2"            8    15.999    2
atom          18    18   C     "bob- C3"            6    12.011    3
atom          19    19   O     "bob- O4"            8    15.999    1

vdw        16       3.900    0.100    0.000
vdw        17       3.405    0.110    0.000
vdw        18       3.780    0.106    0.000
vdw        19       3.300    0.112    0.000

bond       16   17     321.290    1.463
bond       17   18     346.190    1.321
bond       18   18     371.430    1.543
bond       18   19     806.870    1.203

angle      17   16   17      64.630  107.430
angle      16   17   18      80.080  111.750
angle      17   18   18      60.080  111.390
angle      17   18   19      49.910  124.830
angle      18   18   19      59.740  120.360

strbnd     17   16   17      38.000   38.000
strbnd     16   17   18      38.000   38.000
strbnd     17   18   18      18.700   18.700
strbnd     17   18   19      18.700   18.700
strbnd     18   18   19      18.700   18.700

opbend     17   18    0    0      0.000
opbend     18   18    0    0      0.000
opbend     19   18    0    0      0.000

torsion    18   18   17   16   -0.670 0.0 1  6.287 180.0 2  0.000 0.0 3
torsion    19   18   17   16   -0.670 0.0 1  6.287 180.0 2  0.000 0.0 3
torsion    18   17   16   17    0.854 0.0 1 -0.374 180.0 2  0.108 0.0 3
torsion    17   18   18   17   -0.670 0.0 1  6.287 180.0 2  0.000 0.0 3
torsion    19   18   18   17   -0.670 0.0 1  6.287 180.0 2  0.000 0.0 3
torsion    17   16   17   18    0.854 0.0 1 -0.374 180.0 2  0.108 0.0 3
torsion    19   18   18   19   -0.670 0.0 1  6.287 180.0 2  0.000 0.0 3

multipole    16              0.47000
                             0.00000  0.00000  0.00000
                             0.00000
                             0.00000  0.00000
                             0.00000  0.00000  0.00000
multipole    17 18 16       -0.20395
                             0.41415  0.00000  0.48304
                             0.01772
                             0.00000 -0.53194
                             0.40486  0.00000  0.51422
multipole    18 19 17        0.44393
                             0.32971  0.00000  0.49344
                             0.23390
                             0.00000  0.06773
                            -0.35302  0.00000 -0.30163
multipole    19 18 17       -0.60748
                            -0.01630  0.00000 -0.06700
                            -0.35861
                             0.00000  0.19512
                            -0.00995  0.00000  0.16349

polarize        16 1.6000 0.3900 17
polarize        17 0.8320 0.3900 16 18
polarize        18 1.6000 0.3900 17 18 19
polarize        19 0.9140 0.3900 18
```
